# Changes of Host Immunity Mediated by IFN-γ^+^ CD8^+^ T Cells in Children with Adenovirus Pneumonia in Different Severity of Illness

**DOI:** 10.3390/v13122384

**Published:** 2021-11-28

**Authors:** Ruilin Zheng, Yinghua Li, Danyang Chen, Jingyao Su, Ning Han, Haitian Chen, Zhihui Ning, Misi Xiao, Mingqi Zhao, Bing Zhu

**Affiliations:** Center Laboratory, Guangzhou Women and Children’s Medical Center, Guangzhou Medical University, Guangzhou 510120, China; zrl@stu.gzhmu.edu.cn (R.Z.); liyinghua@gzhmu.edu.cn (Y.L.); cdy@stu.gzhmu.edu.cn (D.C.); Sujingyao@stu.gzhmu.edu.cn (J.S.); hanning@gzhmu.edu.cn (N.H.); chenht@stu.gzhmu.edu.cn (H.C.); ningzhihui@stu.gzhmu.edu.cn (Z.N.); xiaomisi@gzhmu.edu.cn (M.X.)

**Keywords:** HAdV, T cells, cytokine, adenovirus pneumonia

## Abstract

The host immunity of patients with adenovirus pneumonia in different severity of illness is unclear. This study compared the routine laboratory tests and the host immunity of human adenovirus (HAdV) patients with different severity of illness. A co-cultured cell model in vitro was established to verify the T cell response in vitro. Among 140 patients with confirmed HAdV of varying severity, the number of lymphocytes in the severe patients was significantly reduced to 1.91 × 10^9^/L compared with the healthy control (3.92 × 10^9^/L) and the mild patients (4.27 × 10^9^/L). The levels of IL-6, IL-10, and IFN-γ in patients with adenovirus pneumonia were significantly elevated with the severity of the disease. Compared with the healthy control (20.82%) and the stable patients (33.96%), the percentage of CD8^+^ T cells that produced IFN-γ increased to 56.27% in the progressing patients. Adenovirus infection increased the percentage of CD8^+^ T and CD4^+^ T cells that produce IFN-γ in the co-culture system. The hyperfunction of IFN-γ^+^ CD8^+^ T cells might be related to the severity of adenovirus infection. The in vitro co-culture cell model could also provide a usable cellular model for subsequent experiments.

## 1. Introduction

HAdV is a double-stranded DNA virus without an envelope. It belongs to the family of Adenoviridae. In 1953, HAdV was first isolated from human adenoid tissue culture by Rowe et al. [1]. Currently, HAdV can be divided into seven species and more than 100 types. Different serotypes of HAdV can cause various diseases in different parts of the body, such as gastroenteritis, conjunctivitis, cystitis, rhinitis, pharyngitis, and diarrhea [2]. Under normal circumstances, most patients infected with HAdV will have mild symptoms and are self-limiting. Although domestic and foreign studies have shown that HAdV is not the main pathogen of adult community-acquired pneumonia, the infection rate and severe disease rate of HAdV in people with low immunity, such as infants and organ transplant patients, are higher than ordinary people [3,4,5]. More importantly, there is currently no specific treatment for adenovirus infection, and severe pneumonia caused by adenovirus infection has a high fatality rate and poor prognosis, which has caused great clinical concern [6]. So far, the pathogenesis between mild and severe illnesses in adenovirus pneumonia patients is still unclear.

In recent years, research has focused on adenovirus as the most widely used carrier in gene medicine, but how the human innate immune system defends HAdV has always been the priority of the research and the difficulty of its application as a vaccine carrier [7,8]. The specific T cell immunotherapy in the immunodeficiency patients and the hematopoietic stem cell transplantation patients is also one of the hotspots of the research [9]. However, the immune mechanisms, such as the immune escape of HAdV, have not yet been clearly studied, and the cellular immune mechanism of specific T cells has not yet been obvious [10,11]. At present, there is still a lack of studies on the immune mechanism of adenovirus infection through changes in the immune system of patients with adenovirus pneumonia. In order to study how the human innate immune system defends against pathogens, some studies compare host immunity in patients with mild and severe illnesses [12,13,14]. These studies indicate that the pathogenesis of different types of respiratory virus infection is related to host immune disorders.

The methods that can predict changes in the acute phase and the development of severe illness have essential values for the treatment and prognosis of adenovirus infection. The understanding of the pathogenesis of adenovirus pneumonia is the basis for the establishment of these methods. It also helps guide the safe application of adenovirus as a vaccine vector. Immune cells, especially lymphocytes, are one of the pivotal parts of the human immune system. The changes in lymphocytes can reflect the status of host immunity in patients with adenovirus pneumonia to a certain extent [15,16]. Thus, the clinical characteristics and T cell function of patients with adenovirus pneumonia in different severity of illness were systematically investigated in this study. The immune response of clinical patients was verified through the co-cultured cell model in vitro.

## 2. Materials and Methods

### 2.1. Inclusion Criteria of the Patients

This study was conducted in Guangzhou Women and Children’s Medical Center (Guangzhou, China) from June 2019 to June 2021. This study was conducted in strict accordance with the current protocols of Guangzhou Women and Children’s Medical Center affiliated with Guangzhou Medical University, and the informed consent of the patients were obtained in the electronic medical system. The study was approved by the Ethics Committee of Guangzhou Women and Children’s Medical Center. The number of ethical review approval documents was 2017021803. A positive nucleic acid testing for HAdV was the direct evidence and the gold standard of the existence of the virus. In this study, patients aged 0–6 years and with positive HAdV real-time PCR results were used as the preliminary inclusion criteria. Furthermore, the clinical symptoms of the patients with positive results of HAdV real-time PCR were used as the final inclusion criteria. According to the electronic medical records (EMR), the included patients were patients with typical respiratory infection symptoms such as irregular fever, cough, and thickening of lung breath sounds during the course of illness.

### 2.2. Grouping Criteria

The adenovirus pneumonia patients were generally classified into two groups with different severity of illness according to the standards for diagnosing and treating children with adenovirus pneumonia (2019 edition). The grouping criteria were as follows: (a) mild illness, the patients with mild illness might have typical clinical symptoms such as irregular fever, cough, and thicker pulmonary breath sounds, as well as the radiological characteristics of the patients with mild illness, which were lung consolidation and mottled fuzzy shadows in the lungs. The patients with mild illness showed one or more typical respiratory symptoms or imaging symptoms during the course of illness. (b) Severe illness, the respiratory rate of the patients with severe illness was significantly accelerated, the infant (age 0–3 years old) exceeded 70 times/min, and the older child (age 3–6 years old) exceeded 50 times/min. Excluding the influences of fever, crying, and other symptoms, the patients with severe illness might have apparent symptoms of cyanosis and dyspnea. Lung consolidation of multiple lung lobes or 2/3 of lung lobes were involved. The patients were accompanied by pleural effusion, with skin oxygen saturation of ≤92%, and combined with other extrapulmonary complications. The patients were in poor basic condition, with the symptoms of refusal to eat and dehydration. The patients had symptoms of confusion. The patients who met any of the above criteria were diagnosed with severe adenovirus pneumonia.

### 2.3. Flow Cytometry Analysis

Heparinized peripheral blood and serum were collected from experimental subjects, and the PBMC was separated from heparinized peripheral blood. The blood samples were obtained when the patients were admitted to the hospital. Furthermore, the serum was separated by centrifugation (1600× *g* for 10 min at 4 °C) and stored at −80 °C until use in the experiments to avoid repeated freezing and thawing. The leukocyte surface CD antigens and cytokine in the cells were stained with fluorescently-labeled monoclonal antibodies, including anti-CD3 (PerCP, 347344), anti-CD4 (BB515, 565996), anti-CD8 (APC-Cy7, 557834), anti-IFN-γ (BV-421, 562988), and anti-IL-8 (BV-510, 563311) from BD Biosciences (New Brunswick, NJ, USA). The detection of the cells and the cytokine were derived from a BD FACSCanto II flow cytometer (BD Biosciences).

### 2.4. Lymphocyte Subsets Counting

The laboratory test results of the patients with adenovirus infection were gathered from EMR to obtain demographic and clinical information. By comparing the absolute and relative counts of lymphocyte subsets between patients with respiratory infections caused by adenovirus in mild and severe illnesses, the status of lymphocytes in patients with different severity of illness was preliminarily analyzed and evaluated. Different subsets of lymphocytes played a crucial role in coordinating the immune response to viral infections. In particular, the decrease in the number of lymphocytes was usually associated with the depletion of lymphocytes, the decrease in proliferation capacity, and the increase in the level of pro-inflammatory cytokines [17,18,19].

### 2.5. Cell Lines and Reagents

MRC-5 cells were purchased from ATCC (#CCL-171, USA). HAdV-7 was separated and cultured at the Center Laboratory (Guangzhou Women and Children’s Medical Center, Guangzhou Medical University, Guangzhou, China). RAD7EGFP was donated by the Guangzhou Institute of Respiratory Disease (Guangzhou, China). Fetal bovine serum (FBS, 10099141C), Dulbecco’s Modified Eagle’s Medium containing 4.5 g/L glucose (DMEM, C11995500BT), Iscove’s Modified Dulbecco’s Medium GlutaMAX (IMDM, 31980030), and PBS (10010049) were bought from Thermo Fisher Scientific (Waltham, MA, USA). Leukocyte Activation Cocktail (550583) and BFA (347688) from BD Biosciences were used in this study.

### 2.6. Cultivation of PBMC

The lymphocyte separation fluid Ficoll (GE, 17-1440-03, Hartford, CT, USA) was used to separate PBMC, which was separated from other components in the fresh anticoagulant blood by density gradient centrifugation. The fresh anticoagulant blood was mixed evenly with PBS in a 1:1 ratio and then slowly added to the liquid surface of the Ficoll. After density gradient centrifugation, lysing solution (BD, 340183, USA) was used to lyse red blood cells, and the PBMC was separated by centrifugation after washing with PBS. Then, the PBMC were then transferred to IMDM containing 10% FBS for culture.

### 2.7. MRC-5 Cells Culture and Virus Infection Titer (TCID_50_) Determination

The MRC-5 cells were cultured in the T25 culture flask at the condition of DMEM containing 10% FBS. When the cell density reached about 70–80%, 1 mL of clinical isolate virus solution was inoculated, and placed in the T25 culture flask (430639, Corning, NY, USA) in a 37 °C, 5% CO_2_ incubator, then observe the cytopathic condition daily. When the cytopathy reached 80–90%, the virus solution was collected and passaged continuously. After two generations of the virus passages, the virus infection titer (TCID_50_) of the collected virus solution was measured on MRC-5 cells and calculated according to the Reed-Muench two-tier method TCID_50_ value. In this experiment, MRC-5 cells were added to a flat-bottomed 96-well cell culture plate at a density of 10^5^ cells/mL, and the growth conditions were 37 °C and 5% CO_2_.

### 2.8. HAdV-7 Infection of MRC-5 Cells by Fluorescence Microscopy Observation

In order to verify that the CPE produced by MRC-5 cells was caused by HAdV-7 infection, rAD7EGFP, a type of HAdV-7 with the eGFP gene inserted, which could produce green fluorescent eGFP when the virus replicated and proliferated was used in the experiment [20,21]. The TCID_50_ of rAD7EGFP was measured on MRC-5 cells and calculated according to the Reed-Muench two-layer method. MRC-5 cells were added to a flat-bottomed 6-well cell culture plate at a density of 1 × 10^5^ cells/mL and grown to account for about 80% of the holes. RAD7EGFP with virulence of 100 TCID_50_/mL was added to the MRC-5 cells and incubated at 37 °C and 5% CO_2_ for 2 h. The MRC-5 cells were washed with PBS to remove the virus that was not internalized. At the moments of 12 h, 24 h, 48 h, and 72 h after rAD7EGFP infection, the CPE condition and fluorescence intensity of the cells were observed by fluorescence microscopy. 

### 2.9. Serum and Culture Supernatant Cytokine Analysis

**Scheme 1:**
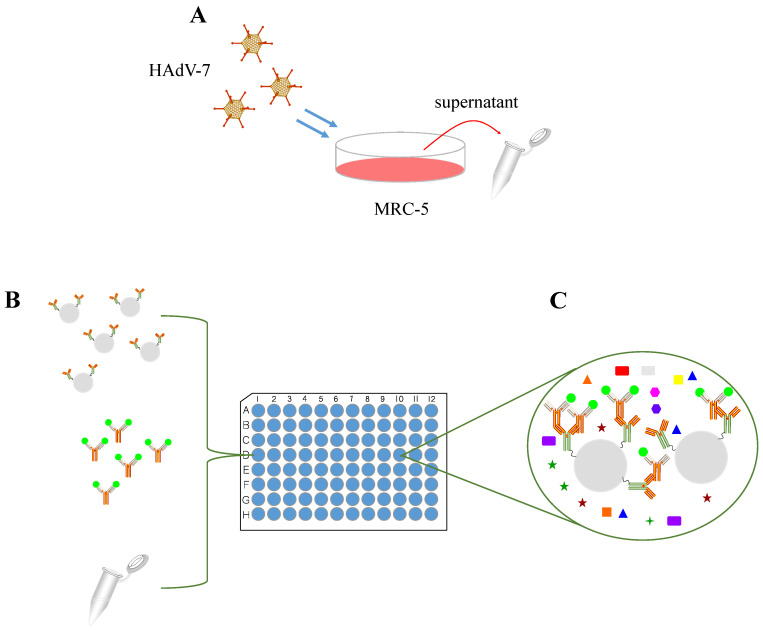
Schematic diagram of culture supernatant cytokine analysis. (**A**) The culture supernatant of MRC-5 cells was extracted at 12 h, 24 h, 48 h, and 72 h. (**B**) The culture supernatant of MRC-5 cells was incubated with the microspheres with cytokine antibodies. (**C**) Flow fluorescence technique and double antibody sandwich method were applied to detect cytokines in the supernatant.

The serum samples and culture supernatant of PBMC were collected from experimental research participants. The levels of IFN-γ, IL-1β, IL-6, IL-8, IL-10, IL-12p70, IL-17F, IL-22, TNF-α, and TNF-β in the serum were measured by the BD FACSCanto II flow cytometer according to the flow immunofluorescence method through Aimplex cytokine detection kit (QuantoBio, Beijing, China). The specific operation process was expressed by Scheme 1. The standard curves of IFN-γ, IL-1β, IL-6, IL-8, IL-10, IL-12p70, IL-17F, IL-22, TNF-α, and TNF-β were determined by the respective standards. The PE fluorescence intensity of the cytokine obtained by FACS was converted into the cytokine levels (pg/mL) through the standard curves, which achieved the purpose of quantitative analysis. FCAP Array v3 was used to analyze the results from the flow cytometer.

### 2.10. T Cell Function

According to previous studies, the function of lymphocytes stimulated by PMA/ionomycin was determined. In this study, 100 μL of heparinized peripheral blood and 100 μL of IMDM medium were evenly mixed. After that, Leukocyte Activation Cocktail, an activation mixture that contained PMA, ionomycin, brefeldin A, and BD GolgiPlug, was used to stimulate the T cells for 4 h. The leukocyte surface CD antigens on the T cells were labeled with monoclonal antibodies (containing anti-CD3, anti-CD4, and anti-CD8). Subsequently, the T cells were fixed and permeabilized. The permeabilized T cells were dyed with intracellular anti-IFN-γ and anti-IL-8 monoclonal antibodies. The results were obtained by flow cytometry, and the results were analyzed by BD FACSDiva software.

### 2.11. T Cell Function Stimulated by Adenovirus

The co-culture system was composed of MRC-5 cells, and PBMC was used in this experiment. MRC-5 cells were added to a flat-bottomed 6-well cell culture plate at a density of 8 × 10^4^ cells/mL, and the growth conditions for MRC-5 cells were 37 °C and 5% CO_2_. When the density of MRC-5 cells reaches 70–80% of the wells, adenovirus with virulence of 100 TCID_50_ was added and incubated at 37 °C and 5% CO_2_ for 2 h. After that, virus fluid was discarded to eliminate the virus that was not internalized, and DMEM containing 1% FBS was added as a maintenance medium. The MRC-5 cells infected by adenovirus were cultured for 48 h under 37 °C and 5% CO_2_. PBMC isolated from adenovirus-infected patients with a density of 10^6^ cells/mL and 10 μg/mL BFA as a protein transport blocker was added to the culture systems of MRC-5 cells and HAdV-7 infected MRC-5 cells. PBMC with 10 μg/mL BFA was added as a blank control group. PBMC mainly included lymphocytes, monocytes, phagocytes, dendritic cells, and a few other cell types. The main objects of this experiment were T cells, which were suspended growth cells [22]. Based on the characteristics of MRC-5 cells growing adherently, and T cells growing in suspension, the adherent growth status of MRC-5 cells was observed through a microscope, and the supernatant of the co-culture system was collected and centrifuged to obtain PBMC. After incubation for 4 h under 37 °C and 5% CO_2_ conditions, PBMC in each group was collected by the above methods. The T cells were marked by monoclonal antibodies of Leukocyte surface CD3, CD4, CD8 antigens, and intracellular anti-IFN-γ and anti-IL-8 monoclonal antibodies. The flow cytometer was used to analyze the T cell function stimulated by adenovirus.

### 2.12. Detection of Cytokine Gene Expression

Most of the cells in PBMC, especially lymphocytes, were suspended growth cells. Through microscopic observation, the MRC-5 cells in the co-culture system maintained adherent growth, which could ensure that most of the cells in the supernatant were PBMC. After confirming the adherent growth of MRC-5 cells, the cell culture plate was gently shaken, and the supernatant of the co-culture system was collected and centrifuged to obtain PBMC. In this experiment, Trizol (#254712, Invitrogen, Thermo Fisher Scientific, USA) was added to lyse the cells for the RNA extraction, and the RNase-Free DNase І system (M6101, Promega, USA) was used to remove genomic DNA. The purity of total RNA was determined by an Eppendorf BioSpectrometer (Eppendorf, Hamburg, Germany) at OD260/OD280. The 5s rRNA, 18s rRNA, and 28s rRNA bands of the total RNA were observed through the gel imaging system after agarose gel electrophoresis to determine the integrity of the total RNA. After confirming that the purity and integrity of the total RNA were good, the total RNA was converted into total cDNA through the process of reverse transcription. The reaction conditions were 30 °C for 10 min, 42 °C for 60 min, and 85 °C for 10 min. The primer design for qPCR was based on the specific conserved coding sequence of the F1 and R1 segments in IFN-γ, IL-8, and RPL-18 cDNA. The primers used for IFN-γ quantification were 5′-GGCAAGGCTATGTGATTACA-3′ and 5′-TAAAGCACTGGCTCAGATTG-3′. The primers used for IL-8 quantification were 5′-GGAAGGAACCATCTCACTGT-3′ and 5′-GGAAGGAACCATCTCACTGT-3′. The primers used for RPL-18 quantification were 5′-CCTGGATACCGCAGCTAGGA-3′ and 5′-GCGGCGCAATACGAATGCCCC-3′, which was used as a standardized control for each gene of PBMC from different groups. SYBR Green qPCR SuperMix (Invitrogen, Thermo Fisher Scientific, USA) was used as a reagent for qPCR quantification. The qPCR was performed on an ABI PRISM® 7500 Sequence Detection System (Thermo Fisher Scientific, USA). The qPCR protocol was: 95 °C for 5 min (1 cycle); 95 °C for 15 s, 60 °C for 32 s (40 cycles); at the end of the 60 °C amplification step, the data of each group were collected. After the amplification was completed, the melting curve analysis was conducted at 60–95 °C.

### 2.13. Statistical Analysis

The experimental data used GraphPad Prism Software, version 8.0, for analysis. The measurement results of each group were manifested as the mean ± SD. A two-tailed Student’s *t*-test was used in the comparison between the two groups, as well as the multiple-group comparisons were performed by one-way ANOVA. When *p* < 0.05 (*), *p* < 0.01 (**), *p* < 0.001 (***), or *p* < 0.0001 (****), the difference was statistically significant.

## 3. Results

### 3.1. General Characteristics of Patients and Healthy Control

According to the primary entry criteria of this study, a total of 140 patients confirmed HAdV with different severity of illness (mild, *n* = 82; severe, *n* = 58) were enrolled. Among the enrolled adenovirus pneumonia patients, the proportion of males was higher than females. From the perspective of diagnostic indicators, the most common clinical symptoms of patients with adenovirus pneumonia were fever (71.4%) and cough (81.4%), and the probability of fever and cough symptoms in severe patients was higher than in mild patients. As the severe patients generally developed acute respiratory distress syndrome, 84.5% of severe patients had symptoms of shortness of breath, and 60.3% of severe patients had hydrothorax. All of the severe patients had more than one clinical symptom, while only 36.6% of mild patients had more than one clinical symptom (Table 1). There was no significant difference in age among the healthy control group (2.88 ± 1.54), the mild group (2.74 ± 1.75), and the severe group (2.65 ± 2.17), as shown in Table 2. According to the routine laboratory tests, some biomarkers had statistical significance in adenovirus pneumonia patients with different severity of illness. For example, the leucocytes number, especially the neutrophils number in mild patients, was significantly increased compared with the healthy control. The lymphocyte number of severe patients was significantly decreased compared with the healthy control. Furthermore, the indicators of total protein, albumin, and alkaline phosphatase of adenovirus pneumonia patients were significantly decreased compared with the healthy control, as well as the indicators of γ-glutamyl transpeptidase and lactate dehydrogenase were significantly increased compared with the healthy control. The indicator of coagulation function such as D-dimer and infection biomarkers, such as procalcitonin, were significantly increased in the patients with severe illness compared with the patients with mild illness. 

### 3.2. Lymphocyte Subsets Counting

**Figure 1:**
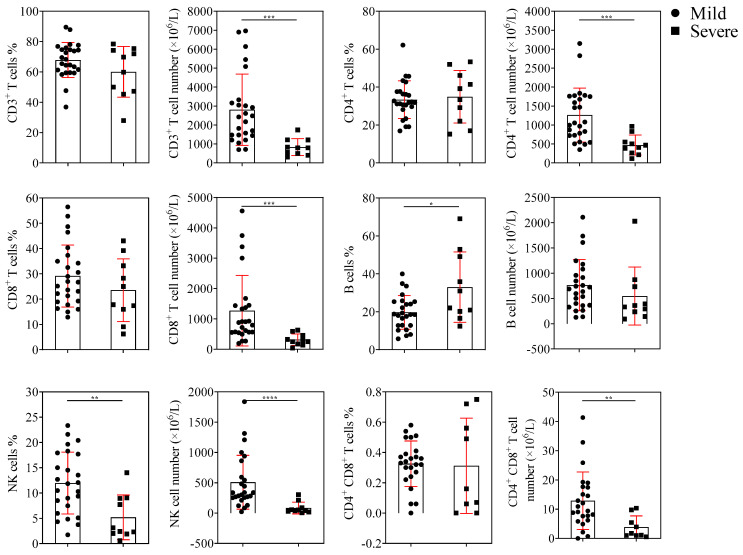
Lymphocyte subsets counting. The percentages and absolute numbers of CD3^+^, CD4^+^, CD8^+^ T cells, B cells, and NK cells in mild (*n* = 25) and severe (*n* = 10) groups are shown (mean ± SD). *p* < 0.05 (*), *p* < 0.01 (**), *p* < 0.001 (***), or *p* < 0.0001 (****) mean the difference was statistically significant.

The EMR showed that the proportion and absolute counts of lymphocyte subsets in healthy individuals were within the normal range. This experiment mainly analyzed the lymphocyte subsets of patients with adenovirus infection. The gender information of patients in the TBNK lymphocytes counting experiment was shown in Table S1. The absolute counts of CD3^+^, CD4^+^, CD8^+^ T cells, and NK cells were significantly reduced in severe patients compared with mild patients. The number of CD4 and CD8 double-positive T cells decreased as the disease progressed in the patients. In contrast, the number of B cells between mild and severe patients showed no significant difference, as shown in Figure 1.

### 3.3. Serum Cytokine Analysis

**Figure 2:**
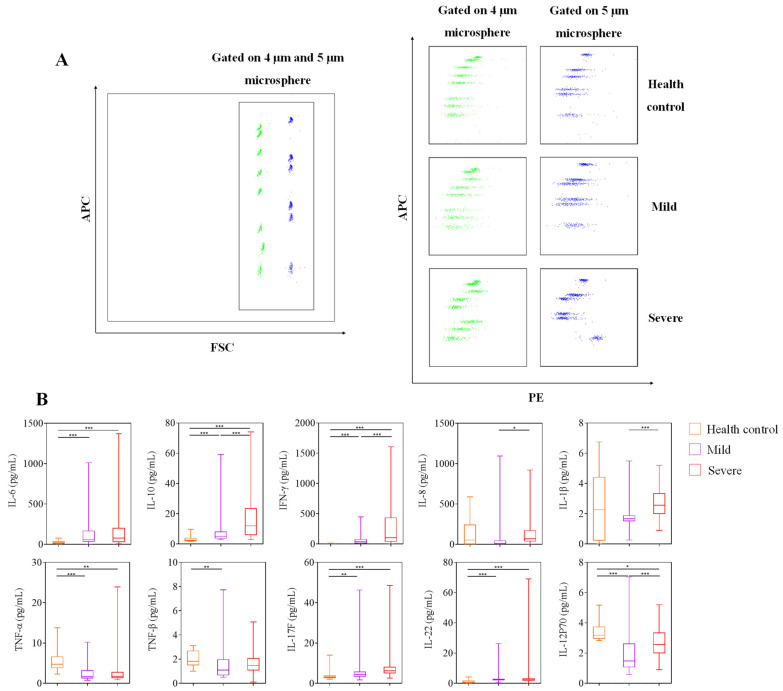
Serum cytokine analysis. (**A**) FACS dot plots showed that cytokine was classified by APC fluorescence, and cytokine levels were detected by PE fluorescence intensity. (**B**) The serum levels of IFN-γ, IL-1β, IL-6, IL-8, IL-10, IL-12p70, IL-17F, IL-22, TNF-α, and TNF-β in the healthy control (*n* = 20), mild (*n* = 82), and severe (*n* = 58) groups shown in graphs (median, IQR). *p* < 0.05 (*), *p* < 0.01 (**) or *p* < 0.001 (***) mean the difference was statistically significant.

**Figure 3:**
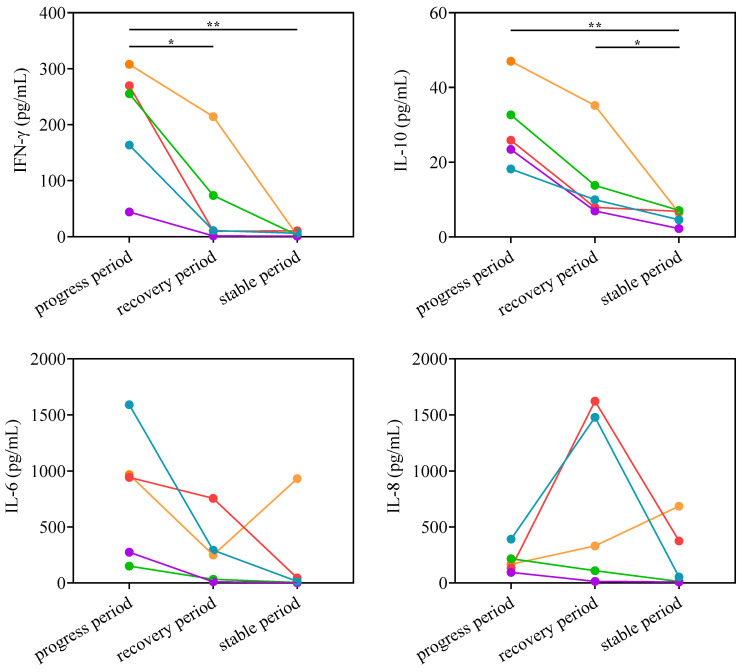
Serum cytokine analysis in severe patients. The serum levels of IFN-γ, IL-10, IL-6, and IL-8 in patients with severe adenovirus pneumonia (*n* = 5) were continuously monitored during the progress, recovery, and stable periods of adenovirus pneumonia. *p* < 0.05 (*) and *p* < 0.01 (**) mean the difference was statistically significant.

The levels of IL-10, IFN-γ, IL-6, IL-17F, and IL-22 in patients with adenovirus pneumonia were significantly higher than those in the healthy control. The levels of IL-10, IFN-γ, IL-8, and IL-1β had a significant difference between mild and severe patients, and the change was gradually increased with increasing severity of illness (Figure 2). According to the above experiment, IL-10, IFN-γ, IL-8, and IL-6 had a high correlation with the patients with severe pneumonia infected by adenovirus. The gender information of patients in the serum cytokine analysis experiment was shown in Table S2. In order to investigate the relationship between the cytokine and the severity of adenovirus pneumonia, five patients were selected from the severe patients, and the complete course of the disease was continuously monitored. Due to the difference in the length of illness between different patients, the disease stage was used to replace the specific time to represent the change of the disease course more effectively. Patients in the progress period showed clinical symptoms of severe pneumonia. Patients in the recovery period showed no obvious fever symptoms and could breathe spontaneously. Patients in the stable period maintained a normal body temperature without respiratory symptoms. The clinical background of the five patients in the continuous monitoring of serum cytokine experiment was shown in Table S3. The serum of the patients was collected during the progress, recovery, and stable period of adenovirus pneumonia, and the cytokine in the serum at different stages of the disease were analyzed. The levels of IL-10 and IFN-γ in severe patients in the progress period were significantly higher than those in the recovery and stable period. As the condition of the patients gradually stabilized, the levels of IL-10 and IFN-γ showed a downward trend (Figure 3). 

### 3.4. Detection of T Cell Function

**Figure 4:**
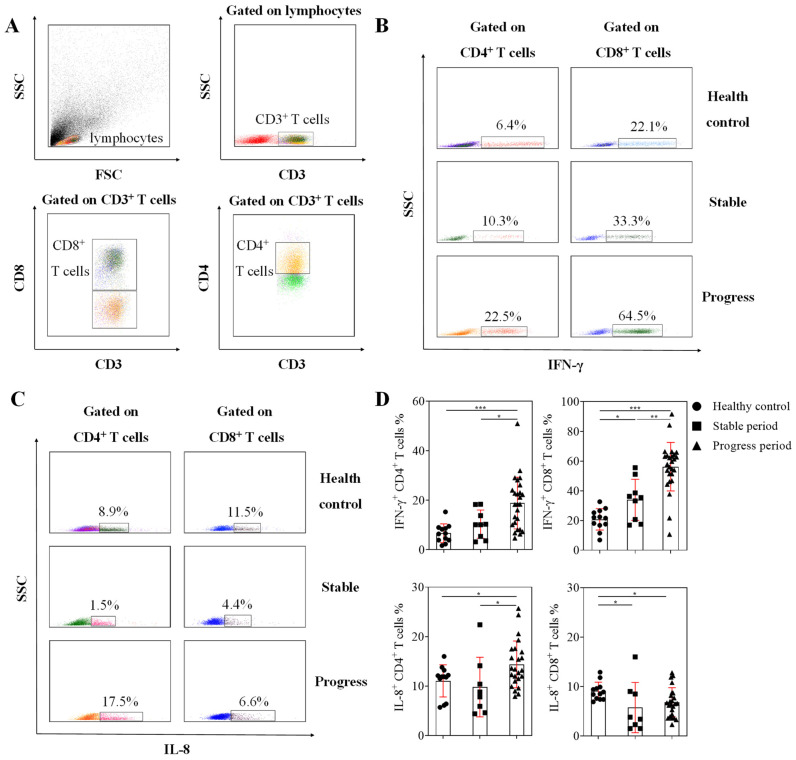
T cell function assay was performed in patients with different periods of illness. (**A**) FACS dot plots showed the classification of T cells. (**B**) FACS dot plots showed the producing ability of IFN-γ in CD4^+^ T cells and CD8^+^ T cells after stimulation. (**C**) FACS dot plots showed the producing ability of IL-8 in CD4^+^ T cells and CD8^+^ T cells after stimulation. (**D**) The percentages of IFN-γ^+^ cells and IL-8^+^ cells in CD4^+^ T cells and CD8^+^ T cells are shown in graphs (mean ± SD). *p* < 0.05 (*), *p* < 0.01 (**), or *p* < 0.001 (***) mean the difference was statistically significant.

The study required a more refined classification of patients with adenovirus pneumonia. In patients with typical clinical symptoms and radiological characteristics, the further classification criteria were as follows: (a) progress period, in which the patients were acute onset, and had high fever above 39 °C at the beginning of the onset. When the patients showed positive adenovirus real-time PCR results, there was a fever accompanied by respiratory symptoms such as coughing and wheezing. (b) Stable period, in which the patients showed positive adenovirus real-time PCR results but continued to maintain a normal body temperature and were without respiratory symptoms such as coughing and wheezing for a week. In order to analyze the T cell function of patients with different disease processes, nine patients in the stable period and 26 patients in the progress period were randomly selected from the enrolled patients. The results of T cell function stimulated by PMA/ionomycin showed that the percentage of IFN-γ producing CD8^+^ T cells in the three groups was higher than that of CD4^+^ T cells, and the IFN-γ producing CD8^+^ T cells in patients with adenovirus pneumonia were significantly higher than the healthy control. The percentages of IFN-γ producing CD8^+^ T and CD4^+^ T cells in progressing patients were significantly higher than those in stable patients and the healthy control. However, there was no difference in the percentage of IFN-γ producing CD4^+^ T cells between patients in stable period and healthy control. The percentage of CD4^+^ T cells that produce IL-8 in progressing patients was significantly higher than that in stable patients and healthy control, but the percentage of CD8^+^ T cells that produce IL-8 in patients with adenovirus pneumonia was lower than that in healthy control, as shown by Figure 4. The results indicated that the hyperfunction of CD8^+^ T cells, which produced IFN-γ, and the hyperfunction of CD4^+^ T cells, which produced IL-8, might be related to the pathogenesis of adenovirus pneumonia.

### 3.5. Effect of HAdV-Infected MRC-5 Cells on Cytokine Secretion

**Figure 5:**
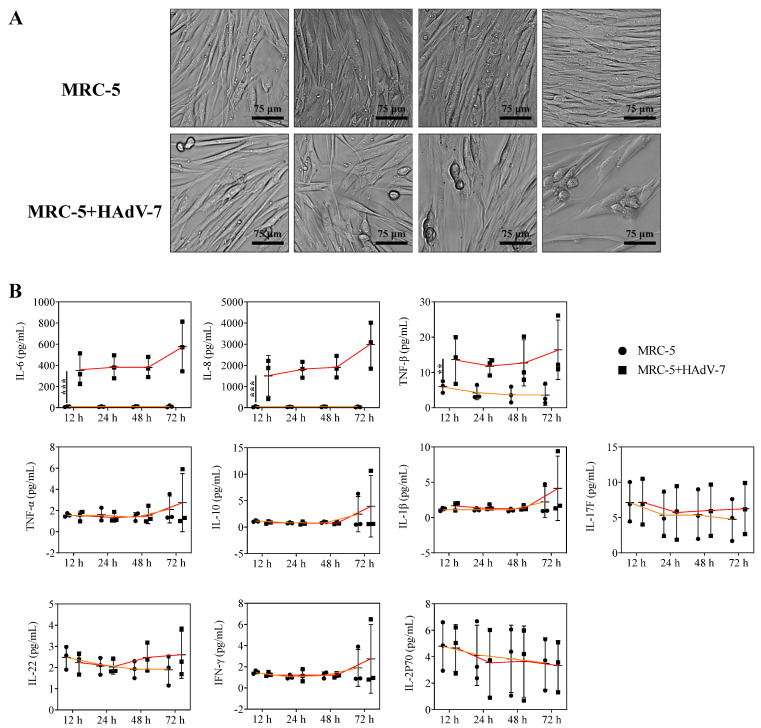
Effect of HAdV-7-infected MRC-5 cells on cytokine secretion. (**A**) The CPE of HAdV-7-infected MRC-5 cells was continuously observed within 12 h to 72 h through an optical microscope. (**B**) The cytokine changes of MRC-5 cells and HAdV-7-infected MRC-5 cells were continuously monitored within 12 h to 72 h through FACS. The results are shown in graphs (mean ± SD), *p* < 0.01 (**), or *p* < 0.001 (***) mean the difference was statistically significant.

The virus virulence test showed that the TCID_50_ of HAdV-7 in MRC-5 cells was 10^4.5^ as listed in Table 3, and HAdV-7 infected the cells with virulence of 100 TCID_50_. The HAdV-7-infected MRC-5 cells were continuously monitored, and the CPE and cytokine changes in MRC-5 cells were recorded from 12 h to 72 h, respectively. HAdV-7-infected MRC-5 cells had noticeable cell pathological change (CPE) and decreased cell number at 48 h and 72 h. There were no differences of TNF-α, IL-10, IL-1β, IL-2P70, IL-17F, IL-22, and IFN-γ in MRC-5 cells compared with HAdV-7-infected MRC-5 cells. However, the levels of IL-6, IL-8, and TNF-β were significantly elevated in MRC-5 cells infected with HAdV-7, and the level of IL-6 and IL-8 showed an upward trend over time (Figure 5).

### 3.6. Observation of HAdV-7 Infected MRC-5 Cells by Fluorescence Microscope

**Figure 6:**
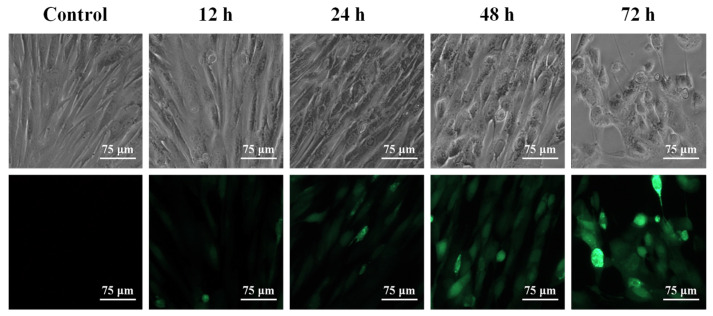
Observation of the CPE and fluorescence intensity of the rAD7EGFP infected MRC-5 cells. MRC-5 cells produced CPE after infection with rAD7EGFP. The cytopathy and the green fluorescence of MRC-5 cells infected with rAD7EGFP gradually increased over time.

The titers of rAD7EGFP are listed in Table 4. MRC-5 cells produced CPE after infection with rAD7EGFP, and the cytopathy gradually increased over time. Interestingly, the green fluorescence of MRC-5 cells infected with rAD7EGFP also gradually increased over time through fluorescence microscope observation, and most of the green fluorescence was expressed on the cells with CPE. The results indicate that HAdV-7 could infect MRC-5 cells, and as the virus replicated and proliferated, the CPE of MRC-5 cells became more severe (Figure 6).

### 3.7. Gene Expression of IFN-γ and IL-8 in the Co-Culture System Composed of MRC-5 Cells and PBMC

**Figure 7:**
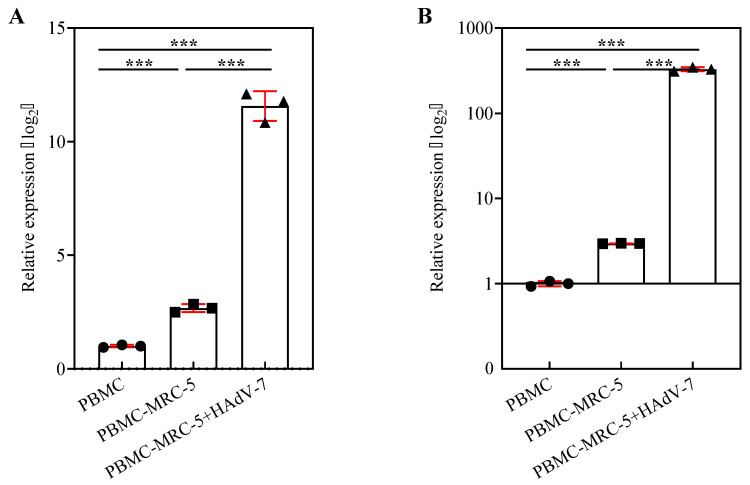
Detection of IFN-γ and IL-8 gene expression of PBMC in a co-culture system. (**A**) IL-8 and (**B**) IFN-γ gene expression were determined by RT-qPCR. The relative gene expressions of IL-8 and IFN-γ in PBMC among group PBMC, group PBMC-MRC-5, and group PBMC-MRC-5 + HAdV-7 were compared. The relative expression was determined by normalizing to RPL-18 expression and then by the 2^−∆∆Ct^ method against group PBMC. The experiment was repeated three times (mean ± SD). *p* < 0.001 (***) means the difference was statistically significant.

According to the results of the above experiments, the lymphocytes of patients with adenovirus infection had an increased ability to produce IL-8 and IFN-γ in the progress period. This experiment utilized the co-culture system composed of MRC-5 cells and PBMC to compare the relative mRNA expression of IL-8 and IFN-γ in PBMC among the group PBMC, group PBMC-MRC-5, and group PBMC-MRC-5 + HAdV-7. Figure 7A,B show that HAdV-7 infection of MRC-5 cells might induce higher IL-8 and IFN-γ expression in PBMC. In particular, the relative expression level of IFN-γ in group PBMC-MRC-5 + HAdV-7 was 382.94, and it was much higher than that in group PBMC (1.00) and group PBMC-MRC-5 (2.96). These data indicated that after the co-culture system composed of MRC-5 cells and PBMC were infected with HAdV-7, the ability of PBMC to produce IL-8 and IFN-γ was enhanced, which was similar to the changes in lymphocytes of clinical patients.

### 3.8. Detection of T Cell Function Stimulated by Adenovirus

**Figure 8:**
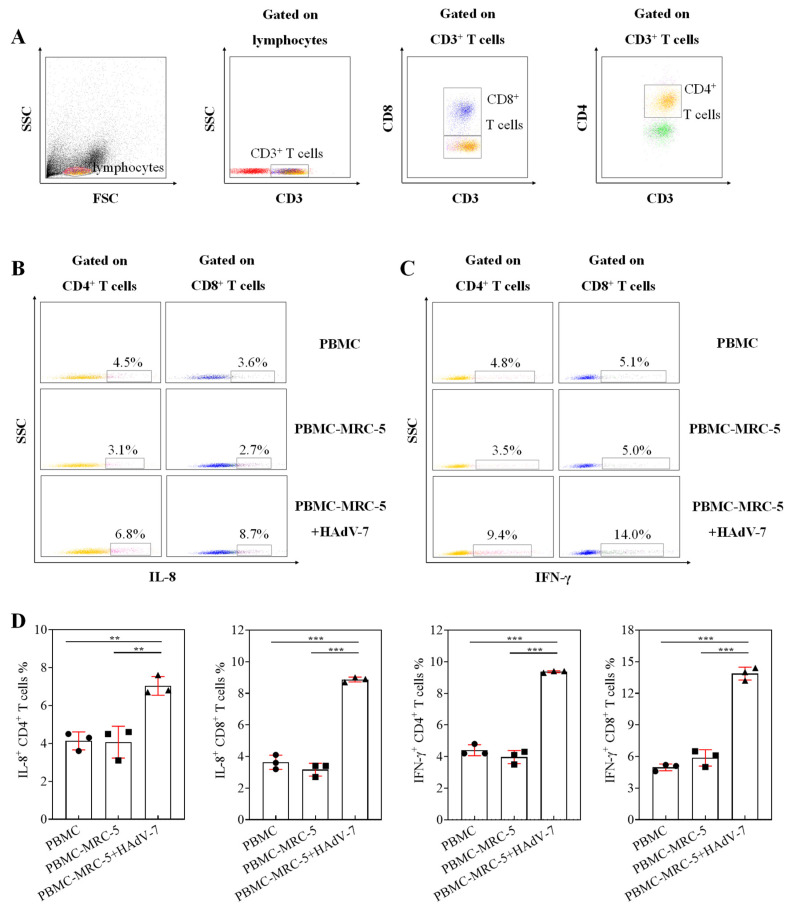
Detection of T cell function stimulated by adenovirus. (**A**) FACS dot plots showed the classification of T cells. (**B**) The FACS dot plots showed the percentage of CD4^+^ T cells and CD8^+^ T cells that produced IL-8 in group PBMC, group PBMC-MRC-5, and group PBMC-MRC-5 + HAdV-7. (**C**) The FACS dot plots shows the percentage of CD4^+^ T cells and CD8^+^ T cells that produced IFN-γ in group PBMC, group PBMC-MRC-5, and group PBMC-MRC-5 + HAdV-7. (**D**) Proportions of T cells producing IL-8 and IFN-γ in each group are shown in graphs. Data expressed as mean ± SD (*n* = 3), *p* < 0.01 (**), or *p* < 0.001 (***) means the difference was statistically significant.

Using the co-culture system composed of MRC-5 cells and PBMC, the percentages of CD8^+^ T and CD4^+^ T cells produced by IFN-γ and IL-8 in group PBMC-MRC-5 + HAdV-7 were significantly higher than those in group PBMC-MRC-5 and group PBMC. In addition, CD8^+^ T cells had a better ability to produce IFN-γ than CD4^+^ T cells in all groups, as demonstrated in Figure 8.

## 4. Discussion

Adenovirus infection has become a challenge facing China and the rest of the world in the field of infant respiratory infections. According to previous studies, most children with adenovirus infection have mild illness and are recovered in about two weeks. However, once infected children develop severe illness characterized by acute respiratory distress syndrome, they may suffer severe lung damage and even death due to multiple organ failures. Since the pathogenesis of adenovirus infection is still unclear, and there is a lack of specific treatment for adenovirus infection, the clinical treatment of patients with adenovirus infection is limited to symptomatic treatment. By analyzing the clinical cases, patients with severe infections usually take anti-inflammatory therapy combined with immunoglobulin for supportive treatment. Therefore, studying the pathogenic mechanism of adenovirus causing respiratory infection has become an urgent clinical need. Through a series of experiments, the most important finding of this study is the hyperfunction of T cells that secrete IFN-γ, especially CD8^+^ T cells, which may be related to the pathogenesis of adenovirus infection.

In this study, we discovered that male infants are more susceptible to adenovirus than females. The most common respiratory symptoms in children infected with HAdV are fever and cough. One of the main signs of patients with adenovirus infection is fever, indicating that the immune system has produced a series of inflammatory responses to adenovirus infection. 

According to previous studies, some routine laboratory biomarkers may exceed the reference range and change with the severity of the disease when the virus is infected [23]. This study found that some biomarkers have different changes in adenovirus pneumonia patients with different severity of illness. For example, the number of white blood cells in mild patients, especially neutrophils, increased significantly, but the number of lymphocytes in severe patients decreased significantly. This result shows that the body activates neutrophils to make an immune response to adenovirus infection, but adenovirus affects the immune system related to lymphocytes. Similar to other viral infections, the indicators of the total protein, albumin, and alkaline phosphatase of adenovirus pneumonia patients are significantly reduced, and indicators of the γ-Glutamyl transpeptidase and lactate dehydrogenase are significantly increased, although there is no direct connection to these biological indicators with adenovirus infection, the results indicate that the circulatory system of adenovirus pneumonia patients is disordered. The indicators of coagulation function, such as D-dimer and infection biomarkers such as PCT in severe patients, were significantly higher than those in mild patients. In previous studies, PCT was used as a sensitive indicator for detecting viral infections, and the level of PCT was correlated with the severity of viral infections to a certain extent [24,25]. This study indicates that the pathogenesis of severe adenovirus infection is related to the decrease of lymphocytes and the disorder of inflammatory response. 

Among the various cells of the immune system, T cells are one of the pivotal elements of the antiviral immune response. Some previous studies have shown that T cells can differentiate into various cell subsets with different functions. During the process of the immune response, they can adjust various physiological and pathological environments adaptively. However, once the function of T cells is dysfunctional, it may cause a disorder of the immune system [26]. Although few studies focus on the relationship between adenovirus pathogenesis and T cells, some comparative studies still show that T cell depletion is related to the mortality of adenovirus-infected hematopoietic cell transplant patients [27,28]. Furthermore, some studies have manifested that adenovirus-specific T cells have a specific antiviral ability against adenovirus [29]. In addition, previous studies have explored the effect of CD8^+^ T cells on the pathogenesis of mouse adenovirus-1 (MAV-1) by establishing an animal model of lung inflammation induced by MAV-1 [30]. The studies have also shown that the immune response of CD8^+^ T cells from mice infected with MAV-1 can promote MAV-1-induced lung inflammation [31]. Benefitted by animal models of MAV-1 infection in mice, some studies indicate that neonates are more susceptible to acute MAV-1 respiratory tract infections than adults. However, they can produce protective CD8^+^ T cell immune responses, and the results of the studies are similar to clinical phenomena [32]. CD8^+^ cytotoxic T cells are the main cells involved in cellular immunity. Cytotoxic T cells have a direct killing effect on antigens and a synergistic killing effect by releasing cytokines, which can effectively protect against the virus infection [33,34,35]. Our study found that one of the typical characteristics of severe adenovirus pneumonia patients is the decrease in the number of lymphocytes, consistent with the results of previous studies [36]. Therefore, we further analyzed the subtypes of lymphocytes and found that the number of CD4^+^ and CD8^+^ T cells decreased as the severity of the disease increased. Meanwhile, the number of NK cells related to cellular immunity in the severe adenovirus pneumonia patients was also significantly reduced. The substantial reduction of lymphocytes and NK cells shows that adenovirus infection may consume immune cells to inhibit cellular immune function.

Previous studies have shown that a vigorous inflammatory response could promote the apoptosis and dysfunction of the T cells [37,38]. Therefore, our study aimed to reflect the state of the immune system by detecting the cytokines in peripheral blood. The experiment results showed that the levels of IL-10, IFN-γ, IL-6, IL-17F, and IL-22 in patients with adenovirus pneumonia increased significantly. As the condition worsened, the levels of IL-10, IFN-γ, and IL-8 gradually increased. The levels of IL-10 and IFN-γ in severe progressive patients increased significantly. As the condition of the patients gradually stabilized, the levels of IL-10 and IFN-γ showed a downward trend. We found that the increase of IL-10 and IFN-γ had certain relevance with changes in the course of adenovirus pneumonia. According to previous studies, IL-6 is mainly secreted by macrophages and B cells and plays a role in regulating the differentiation and activation of T cells. IL-8 was mainly secreted by activated monocytes and macrophages. In addition, lymphocytes, neutrophils, fibroblasts, endothelial cells, and epithelial cells all had a certain ability to secrete IL-8. IL-6 and IL-8 take part in the generation of inflammation and participate in the regulation and dysregulation of the immune system in many diseases [39,40,41]. Some of the anti-inflammatory cytokines such as IL-10 have a negative feedback regulation to Th1 cells, NK cells, and macrophages to effectively reduce the damage caused by inflammation, and hinder the removal of pathogens during infection. The increased level of IL-10 in patients with severe adenovirus pneumonia may be related to the immune escape mechanism of the virus [42]. Through the analysis of the experiments, we speculate that the decrease in peripheral blood lymphocytes and the increase in IL-10 levels may be due to the imbalance of cytokines and the migration of lymphocytes caused by viral infection. According to previous studies, IFN-γ is most commonly used to reflect the virus infection process, and IFN-γ reflects the ability to clear the virus to a certain extent [43,44,45]. IFN-γ reduces progeny virus production by inhibiting the DNA replication of adenovirus, and the gene products of adenovirus also have an antagonistic effect on IFN-γ. Therefore, as an important cytokine for antiviral and virus escape mechanisms, IFN-γ has become the focus of our research.

By detecting T cell functions, we discovered that the ability of T cells in patients with adenovirus pneumonia to produce IFN-γ increased, which also shows that T cells were hyperactive. In each stage of adenovirus infection, CD8^+^ T cells are the central T cells that secrete IFN-γ, and the activation of CD8^+^ T cells further enhance the immune response. The macrophage activation syndrome may be triggered by the hyperfunction of CD8^+^ T cells, which may lead to immune system disorders and cytokine storms in patients with adenovirus pneumonia.

The human embryonic fibroblast cell line (MRC-5 cell) was a normal human diploid cell and one of the most important cell lines for the preparation of viral vaccines. In addition, adenovirus pneumonia was mainly affected by lung interstitium, and fibroblasts account for 35–40% of the interstitial lung cells [46]. According to the preliminary experiments, we found that MRC-5 could show obvious CPE after HAdV-7 infection, as shown in Figure 5. Therefore, MRC-5 was judged to be a susceptible cell of HAdV-7. In order to determine the relationship between pulmonary adenovirus infection and the secretion of cytokine, the MRC-5 cell was selected to be the target cell in this study. According to previous research and electronic medical records, HAdV-7 has a high incidence in children and can cause serious complications. Epidemiological reports indicate that HAdV-7 has a strong correlation with severe or fatal adenovirus diseases in children. Thus, HAdV-7 was used in this study [47,48]. The experimental results showed that the levels of IL-6 and IL-8 in MRC-5 cells infected with HAdV-7 increased significantly and showed an upward trend over time. MRC-5 cells effectively mimic the cytokine changes that occur after primary lung infection with HAdV to a certain extent. Due to the virus infection, MRC-5 cells mainly secrete IL-6, IL-8, and other inflammatory factors, which may be related to the lung inflammation damage during the infection of HAdV. According to the detection of gene expression of IFN-γ and IL-8 and T cell function of PBMC in vitro, the co-culture system composed of MRC-5 cells and PBMC model was able to reflect the immune response, especially cellular immunity in clinical patients.

**Figure 9:**
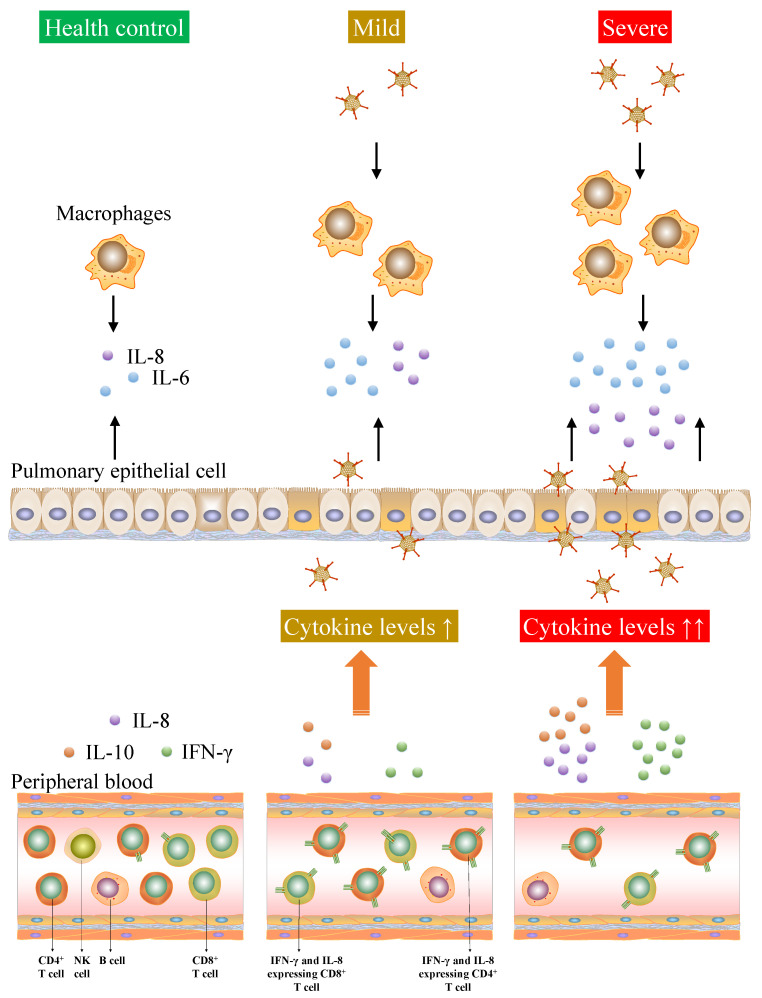
Cytokine storm and the lymphopenia of T cells were associated with the severity of adenovirus infection.

Previous studies have shown that virus-specific T cell responses are essential for controlling virus replication and preventing chronic infectious diseases. In viral infections, protective antiviral immunity is related to IFN-γ secretion, virus-specific CD4^+^ and CD8^+^ T cell responses [49]. We speculate that the adenovirus infection of MRC-5 cells leads to CPE and induces the cells to secrete a series of inflammatory factors such as IL-6 and IL-8. The viral antigens and the inflammatory factors may stimulate PBMC to secrete IFN-γ and other cytokines to resist viral infections. The results of the co-culture system composed of MRC-5 cells and the PBMC model were able to reflect the function of T cells in clinical patients to a certain extent, both of the clinical and in vitro experiments highlighted that IFN-γ producing CD8^+^ T cells played a key role in the immunity of adenovirus infection (Figure 9). 

In recent years, research on adenoviruses has mainly focused on their application as vaccine vectors, but they can induce innate and adaptive immune responses in mammalian hosts. The cellular immune mechanism of adenovirus-infected T cells, especially the immune response involved in T cells, is not yet fully understood. This study systematically investigated the clinical and cellular immune characteristics of children with adenovirus pneumonia of different severity. Compared with mild patients, the number of peripheral blood lymphocytes in children with severe adenovirus infection decreased significantly, especially the absolute counts of CD3^+^, CD4^+^, CD8^+^, and NK cells. It indicated that the cellular immunity in severe patients might be suppressed by adenovirus. In order to investigate the relationship between the cytokine and severity of adenovirus pneumonia, five severe patients with the complete course of the disease were selected. We found that the increase of IL-10 and IFN-γ in serum has certain relevance with changes in the course of adenovirus pneumonia. As the condition of the patients gradually stabilized, the levels of IL-10 and IFN-γ showed a downward trend. In this study, MRC-5 cells were used as the target cells for adenovirus infection to investigate the relationship between adenovirus infection and secretion of cytokine. HAdV-7 infected MRC-5 cells showed obvious cell pathological change. The level of IL-6, IL-8, and TNF-β were significantly elevated in MRC-5 cells infected with HAdV-7, and the level of IL-6 and IL-8 showed an upward trend over time. The co-culture system composed of MRC-5 cells and PBMC model was able to reflect the function of T cells in clinical patients to a certain extent. Both of the clinical and in vitro experiments highlighted that IFN-γ-producing CD8^+^ T cells played a key role in the immunity of adenovirus infection. The in vitro co-culture cell models could provide a usable cellular model for subsequent experiments. Through a series of experiments, the most important finding of this study was the hyperfunction of T cells that secreted IFN-γ, especially CD8^+^ T cells, which might be related to the pathogenesis of adenovirus infection.

## Figures and Tables

**Table 1:** The demographic and clinical characteristics of 140 patients with HAdV infection.

	Mild (*n* = 82)	Severe (*n* = 58)
Age (mean ± SD), years	2.74 ± 1.75	2.65 ± 2.17
Sex		
Male	54 (65.9%)	39 (67.2%)
Female	28 (34.1%)	19 (32.8%)
Signs and symptoms at diagnosis		
Fever	50 (61.0%)	50 (86.2%)
Cough	60 (73.2%)	54 (93.1%)
Shortness of breath	1 (1.2%)	49 (84.5%)
Hydrothorax	-	35 (60.3%)
Confusion	-	6 (10.3%)
More than 1 sign or symptom	30 (36.6%)	58 (100%)

**Table 2:** The biomarkers in adenovirus pneumonia patients with different severity of illness.

	Healthy Control (*n* = 20)	Mild (*n* = 82)	Severe (*n* = 58)
Age (mean ± SD), years	2.88 ± 1.54	2.74 ± 1.75	2.65 ± 2.17
Blood routine			
Leucocytes (1 × 10^9^/L)	7.90 (2.01)	13.91 (6.89) ****	6.43 (4.62) *
Increased	0	43 (52%)	3 (5%)
Decreased	1 (5%)	4 (5%)	27 (47%)
Neutrophils (1 × 10^9^/L)	3.20 (1.57)	8.35 (5.93) ****	4.05 (3.36)
Increased	0	40 (49%)	7 (12%)
Decreased	6 (30%)	8 (10%)	15 (26%)
Lymphocytes (1 × 10^9^/L)	3.92 (1.26)	4.27 (2.08)	1.91 (1.55) ****
Increased	4 (20%)	27 (33%)	5 (9%)
	0	4 (5%)	36 (62%)
Blood biochemistry			
Total protein (g/L)	70.60 (3.46)	67.36 (6.95) *	60.96 (8.81) ****
Decreased	1 (5%)	22 (27%)	41 (71%)
Albumin (g/L)	46.91 (2.01)	39.79 (6.10) ****	29.19 (4.55) ****
Decreased	0	37 (45%)	56 (97%)
Total bilirubin (μmol/L)	5.06 (2.09)	4.52 (4.14)	5.63 (7.16)
Increased	0	1 (1%)	1 (2%)
Decreased	0	5 (6%)	3 (5%)
γ-Glutamyl transpeptidase (U/L)	10.24 (2.34)	13.67 (9.21) **	47.53 (76.18) ****
Increased	0	2 (2%)	13 (22%)
Decreased	7 (35%)	12 (15%)	3 (5%)
Alkaline phosphatase (U/L)	220.65 (43.12)	180.59 (57.66) ***	102.09 (52.63) ****
Decreased	1 (5%)	9 (11%)	38 (66%)
Lactate dehydrogenase (U/L)	256.17 (36.51)	349.09 (215.90) *	1375.10(906.88) ****
Increased	1 (5%)	28 (34%)	54 (93%)
Blood uric acid (mmol/L)	272.21 (80.12)	260.19 (83.92)	213.16 (128.64) **
Increased	2 (10%)	3 (4%)	5 (9%)
Decreased	1 (5%)	0	5 (9%)
Coagulation function			
D-dimer (μg/mL)	-	0.68 (1.21)	3.74 (5.68) ****
Increased	-	4 (5%)	26 (45%)
Infection biomarkers			
Procalcitonin (ng/mL)	-	1.20 (4.09)	12.31 (23.93) ****
Increased	-	34 (41%)	46 (79%)
C-reactive protein (mg/L)	-	28.93 (32.88)	37.69 (41.97)
Increased	-	45 (55%)	47 (81%)
Radiological characteristics			
Thickened lung texture	-	81 (99%)	58 (100%)
Mottled fuzzy shadows	-	37 (45%)	56 (97%)

Data are presented by n (%) and mean (±SD). An increase mean over the upper limit of the normal range, and a decrease mean below the lower limit of the normal range. *p* < 0.05 (*), *p* < 0.01 (**), *p* < 0.001 (***), or *p* < 0.0001 (****), which mean healthy control vs. mild or healthy control vs. severe had statistical difference.

**Table 3:** Titer of HAdV-7.

Dilution of HAdV-14	CPE (Wells)	No CPE (Wells)	Accumulation		Ratio of CPE (%)
			CPE (Wells)	No CPE (Wells)	
10^−1^	8	0	32	0	100
10^−2^	8	0	24	0	100
10^−3^	8	0	16	0	100
10^−4^	8	0	8	0	100
10^−5^	0	8	0	8	0
10^−6^	0	8	0	16	0
10^−7^	0	8	0	24	0
10^−8^	0	8	0	32	0

The Reed-Muench assay was used to test the titer of HAdV-7 (TCID_50_ = 10^4.5^).

**Table 4:** Titer of rAD7EGFP.

Dilution of HAdV-14	CPE (Wells)	No CPE (Wells)	Accumulation		Ratio of CPE (%)
			CPE (Wells)	No CPE (Wells)	
10^−1^	8	0	28	0	100
10^−2^	8	0	20	0	100
10^−3^	8	0	12	0	100
10^−4^	4	4	4	4	50
10^−5^	0	8	0	12	0
10^−6^	0	8	0	20	0
10^−7^	0	8	0	28	0
10^−8^	0	8	0	36	0

The Reed-Muench assay was used to test the titer of rAD7EGFP (TCID_50_ = 10^4^).

## Data Availability

The data presented in this study are available in the article.

## Supplementary Materials

The following are available online at https://www.mdpi.com/article/10.3390/v13122384/s1: Table S1. The gender of patients in the TBNK lymphocytes counting experiment; Table S2. The gender of patients in the serum cytokine analysis experiment; Table S3. The clinical background of the patients in the continuous monitoring of serum cytokine experiment.

## Abbreviations

HAdV: human adenovirus; PCT, procalcitonin; EMR, electronic medical records; TCID_50_, median tissue culture infective dose; PMA, phorbol myristate acetate; CPE, cell pathological change.
