# Supplementary material for: Changes of Host Immunity Mediated by IFN-γ+ CD8+ T Cells in Children with Adenovirus Pneumonia in Different Severity of Illness
Source: Viruses. 2021 Nov 28;13(12):2384. doi: 10.3390/v13122384 (PMC8708941; doi:10.3390/v13122384)
Supplement: Supplementary file 1 [file viruses-13-02384-s001.zip › viruses-1438287-supplementary.pdf]

Supplementary data

The gender information of patients in the TBNK lymphocytes counting experiment was shown in Supplementary Table S1.

Table S1. The gender of patients in the TBNK lymphocytes counting experiment

	Mild (n = 25)	Severe (n = 10)
**Age (mean ± SD), years**	2.73 ± 1.59	2.05 ± 0.93
**Sex**		
Male	17 (68.0%)	4 (40.0%)
Female	8 (32.0%)	6 (60.0%)

The gender information of patients in the serum cytokine analysis experiment was shown in Supplementary Table S2.

Table S2. The gender of patients in the serum cytokine analysis experiment

	Health control (n = 20)	Mild (n = 82)	Severe (n = 58)
**Age (mean ± SD), years**	2.88 ± 1.54	2.74 ± 1.75	2.65 ± 2.17
**Sex**			
Male	12 (60.0%)	54 (65.9%)	39 (67.2%)
Female	8 (40.0%)	28 (34.1%)	19 (32.8%)

Most of the gender information of patients with adenovirus infection in each group of the experiments showed that the proportion of males was higher than females, except for the severe group of TBNK lymphocytes counting experiment.

The clinical background of the five patients in the continuous monitoring of serum cytokine experiment was shown in Supplementary Table S3.

Table S3. The clinical background of the patients in the continuous monitoring of serum cytokine experiment

	Severe adenovirus pneumonia patients (n = 5)	Normal range of the clinical indicators
**Age (mean ± SD), years**	1.42 ± 0.61	-
**Sex**		
Male	4 (80%)	-
Female	1 (20%)	-
**Signs and symptoms at diagnosis**		
Fever	5 (100%)	-
Cough	5 (100%)	-
Shortness of breath	5 (100%)	-
Hydrothorax	5 (100%)	-
**Blood routine**	-	
Leucocytes (1×10^9/L)	3.20 (2.70, 4.00)	5.00-12.00
Neutrophils (1×10^9/L)	1.97 (1.48, 2.45)	2.00-7.20
Lymphocytes (1×10^9/L)	1.29 (1.13, 1.61)	1.55-4.80
**Lymphocyte subsets counting**		
$CD3^+$ T cells (%)	49.00 (47.31, 50.70)	50.00-84.00
$CD3^+$ T cells number (1×10^6/L)	735.45 (684.16, 854.06)	690.00-2540.00
$CD4^+$ T cells (%)	23.39 (21.70, 36.21)	30.00-60.00
$CD4^+$ T cells number (1×10^6/L)	395.41 (368.40, 508.16)	410.00-1590.00
$CD8^+$ T cells (%)	18.80 (12.82, 26.20)	13.00-41.00
$CD8^+$ T cells number (1×10^6/L)	297.78 (178.30, 468.82)	190.00-1140.00
B cells (%)	45.57 (40.27, 46.15)	5.00-18.00
B cells number (1×10^6/L)	660.14 (589.40, 665.41)	90.00-660.00
NK cells (%)	1.51 (0.76, 3.97)	7.00-40.00
NK cells number (1×10^6/L)	19.50 (9.49, 57.56)	90.00-590.00

Because the data skew coefficient of most patients' clinical indicators was less than -1 or greater than 1, median (IQR: 1st, 3rd quartiles) was used to present the data. The 5 patients selected for this experiment all had clinical symptoms such as fever, cough, shortness of breath, and hydrothorax. At the same time, the leucocytes, especially lymphocytes were below the normal range in most patients.
